# Multimodal social context modulates larval behavior in *Drosophila*

**DOI:** 10.1126/sciadv.ady0750

**Published:** 2026-01-30

**Authors:** Akhila Mudunuri, Élyse Zadigue-Dubé, Katrin Vogt

**Affiliations:** ^1^ Department of Biology, University of Konstanz, Konstanz, Germany.; ^2^ Centre for the Advanced Study of Collective Behaviour, University of Konstanz, Konstanz, Germany.; ^3^ Max Planck Institute of Animal Behavior, Konstanz, Germany.; ^4^ International Max Planck Research School for Quantitative Behaviour, Ecology and Evolution, Konstanz, Germany.; ^5^ Department of Biology, McGill University, Montréal, Quebec, Canada.

## Abstract

All animals need to navigate and make decisions in social environments. They influence each other’s behavior, but how important this is and how they process and represent social information in their brain are less well understood. This includes fruit flies and larvae, usually not known as “social insects.” Using a *Drosophila* larva assay with reduced stimulation, we found that groups show enhanced dispersal and distance from each other in the absence of food. This social context–dependent modulation overrides responses to many external sensory cues and is shaped by developmental social experience. Leveraging the genetic toolbox available in *Drosophila*, we find that different sensory modalities are required for social context modulation. Our results show that even less social animals such as fly larvae are affected by conspecifics and recognize each other through multimodal sensory cues. This study provides a tractable system for future dissection of the neural circuit mechanisms underlying social interactions.

## Teaser

The social life of fly larvae is complex, shaped by conspecific exposure during development, and influences decision-making.

## INTRODUCTION

Navigating social interactions is essential in any individual’s life and can have strong implications for fitness and survival. Living with conspecifics can be advantageous, as it allows for shared and better access to environmental information, facilitating access to resources (*1*, *2*) or improving predator avoidance (*3*, *4*). However, group living also increases competition for mates and limited food sources (*5*, *6*). Therefore, decision-making in a social context, in interactions with other animals and conspecifics, is complex and requires the integration of all available information from the environment. Social context is not only omnipresent and critical for decision-making but is also required for healthy development (*7*). As the brain evolved in a social world, further investigation into the social neural circuits is necessary to understand its full functionality.

Understanding how conspecifics sense each other is a good starting point for understanding social behavior from a neuroscience perspective. In locusts, mechanosensory interactions play a crucial role in swarm formation (*8*, *9*), mosquitoes integrate visual and acoustic information to swarm (*10*), and ants rely on both mechanosensation and chemosensation to communicate (*11*). Zebrafish primarily seem to use vision to interact with conspecifics (*12*), whereas pheromones trigger sexual or aggressive behaviors in mammals (*13*). Despite these insights, we know little about the underlying neural circuitry that processes this sensory information. Studying social behavior in *Drosophila* is promising, as the available genetic toolkit and the available whole-brain connectome make it a tractable system for dissecting the circuit mechanisms underlying conspecific interactions.

*Drosophila melanogaster*, although not considered a social insect, is exposed to conspecifics across all developmental stages. Flies exhibit local aggregation even in the absence of external stimuli (*14*–*16*) and aggregate on food to mate and oviposit (*17*, *18*). Similarly, *Drosophila* larvae socially aggregate to dig together into food substrate to reach better resources (*19*). This collective digging occurs during their feeding stage; however, when entering the wandering state to look for a pupation site, aggregation behavior decreases (*20*). Larval aggregation can also be beneficial, as they can collectively defend themselves against a harmful fungus (*21*). However, when food is scarce or under extreme crowding, they need to compete for resources and can even resort to cannibalism (*22*). Thus, flies and fly larvae face many complex decisions when interacting with conspecifics and need to integrate social information.

In flies, multisensory cues mediate social interactions. Mechanosensory neurons are necessary to convey social information to enhance a collective odor avoidance response (*23*). The formation of spontaneous fly clusters in the absence of external stimuli requires multiple sensory modalities such as vision, audition, olfaction, taste, and touch (*15*). Flies can learn to become sociable by recognizing conspecific smell and integrating this information within the mushroom body (*24*). How larvae sense each other and how larval-larval interactions are affected by changes in the environment or prior social experience are less studied. Larvae require vision to coordinate collective digging, to be attracted to moving conspecifics, and to avoid collisions (*19*, *25*, *26*). Collective digging further requires the mechanosensory channel NompC (No mechanoreceptor potential C) (*19*). Larval deposits induce attraction via the ppk23 (pickpocket 23) receptor (*27*). However, larvae suppress cannibalism toward fly eggs coated with a maternal pheromone, which is also sensed by the ppk23 receptor (*28*). Thus, larval social interactions are complex, context dependent, and involve multiple sensory systems.

In this study, using a well-controlled and stimulus-reduced behavioral assay, we show that fly larvae disperse away from conspecifics in the absence of any external stimuli. This robust avoidance behavior is prioritized over responses to several other external stimuli, such as aversive light and attractive fructose. During development, larvae adapt to social context, as larvae raised in isolation from the egg show increased dispersal from conspecifics. Investigating the sensory systems involved in conspecific avoidance, we show that multiple sensory modalities are required for normal social recognition and avoidance behavior. Our results highlight the importance of social context on behavior and the complexity of social sensory cues provided by conspecifics.

## RESULTS

### *Drosophila* larvae disperse away from conspecifics

**Fig. 1.**
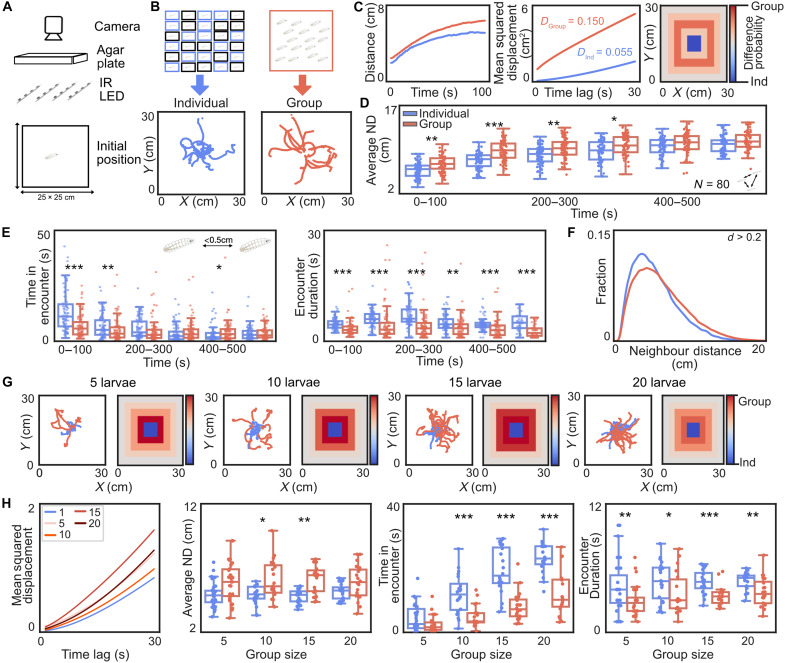
*Drosophila* larvae in a group show enhanced dispersal. (**A**) Larvae were placed in the center of a square plate (25 by 25 cm) containing 2% agar and recorded for 10 min with infrared illumination. IR, infrared. (**B**) Individuals or groups of 15 larvae were tested. Tracks of 15 individuals were superimposed to form a control group. Sample trajectories for the first 100 s of an experiment for superimposed (blue) and real group (red). (**C**) Average distance from the center was plotted for the first 100 s for superimposed groups (blue) and real groups (red). MSD for all real and superimposed groups (*N* = 80 trials per treatment) was plotted as a function of time interval for the first 100 s. The heatmap visualizes the spatial distribution of larvae for the first 100 s by subtracting the probability of finding an individual larva from a real group larva. Blue indicates an abundance of individual larvae, and red indicates an abundance of real group larvae. (**D**) The average neighbor distance (AND) between all pairs in a real or superimposed group over time is shown. (**E**) Real group larvae spent less time in encounters than larvae in the superimposed group. (**F**) Neighbor distances (NDs) for the first 100 s in superimposed (blue) and real (red) groups [effect size = Cohen’s *d* (0.2 < *d* < 0.5: small)]. (**G**) Trajectories and heatmaps of larval groups of 5 (*N* = 30), 10 (*N* = 20), 15 (*N* = 20), and 20 (*N* = 20) animals compared to superimposed individual groups with the same sizes. (**H**) MSD and ND of all group sizes for the first 100 s. Time in encounter and encounter duration of real group larvae are lower across all tested group sizes. [Bootstrapped confidence interval (CI) test = *P < 0.05, **P < 0.01, and ***P < 0.001].

To understand larval-larval interactions, we tested groups of 15 wild-type (WT) Canton-S larvae in a square arena (25 cm by 25 cm) containing 2% agarose in the dark for 10 min (Fig. 1A and movie S1). Larvae started in the middle of the arena, and their behavior was recorded and analyzed using the custom tracking software TRex (movie S2) (*29*). As a control for biases in the arena (border preference) and locomotion defects, we also tested individual larvae and randomly assigned 15 unique individuals into a superimposed individual group for further analysis (Fig. 1B and movie S3). We analyzed the first 100 s of the experiment in detail to study their dispersal behavior. Individual larvae tend to stop more and make bigger turns compared to the group (Fig. 1B and fig. S1, A and B). Larvae in the group move further away from the center compared to individual larvae (Fig. 1C). Analysis of the mean squared displacement (MSD) from the center shows that larvae in the group disperse more than individual larvae over the whole time of the experiment (Fig. 1C and fig. S1C). We visualized larval distribution of all tested animals in a heatmap by plotting the difference in probability of finding a grouped larva to an individual larva in a certain arena location (Fig. 1C). There are more individuals in the middle of the arena (blue squares), whereas grouped larvae are more likely to be found away from the center (red squares). This trend also persists over the whole experiment as seen in both heatmaps and distance from center (fig. S1, D and E). We asked whether larvae in groups generally disperse in the arena, even when presented with a food context. When adding fly food to the arena center, the group, however, stays in the center (movie S4 and fig. S1F).

To quantify social interactions between larvae in the empty arena, such as clustering, we analyzed average neighbor distance (AND), the average distance between all larval pairs in superimposed individual groups and real groups of 15 larvae. Over 10 min, the AND of the real group steeply increases and plateaus after a few minutes (Fig. 1D).

In the superimposed individual group, the AND slowly and gradually increases over 10 min. Overall, the AND of the real group is significantly higher than the AND of the superimposed individual group. This is consistent over the first 200 s for most combined WT experiments in this dataset (fig. S1G). To quantify how larvae interact locally, we counted frames with close encounters of less than 0.5 cm of distance between two larvae (Fig. 1E). This time in encounters and their durations are lower in the real group compared to the superimposed individual group. Only larvae in real groups pause during an encounter and move straight away afterward (fig. S1H). Larvae in the superimposed individual group with no real encounters show no change in behavior. Larvae start in the center and thus encounter conspecifics most often in the first 100 s (Fig. 1E). Within this time frame, neighbor distances (NDs) are higher in the real group, supporting further that grouped larvae do not cluster but spread out evenly to avoid conspecifics (Fig. 1F). Over the whole experiment, the NDs in the superimposed individual group become more similar to the values of the real group (fig. S1I), possibly due to arena size limitations.

We hypothesized that grouped larvae might disperse more in the first 100 s due to collisions with conspecifics. Therefore, we tested whether obstacle collision would arouse larvae and lead to dispersal. We added small agar pieces to the arena and tested individuals (movie S5) and groups (fig. S1J). We found that larvae are rather attracted to the obstacles and disperse less than the control, especially when tested individually. Thus, only the encounter with a conspecific and not just any collision enhances dispersal.

We find similar social context modulation when testing groups of different sizes (Fig. 1G; 5, 10, 15, and 20 larvae). The MSD and AND in all groups are higher than in the individuals (Fig. 1H). Conspecific encounters in all real groups are shorter than in the superimposed individual groups of the same size (Fig. 1H). All groups were tested in the same arena, and thus, the available space for larvae to spread out is limited in bigger group sizes. We, therefore, chose a group size of 15 for all further experiments.

### Social context modulation overrides responses to several external stimuli

Social context modulates the behavior of larvae in an empty experimental arena. As groups rarely interact in an unstimulated environment, we next exposed larvae to two different external stimuli.

**Fig. 2.**
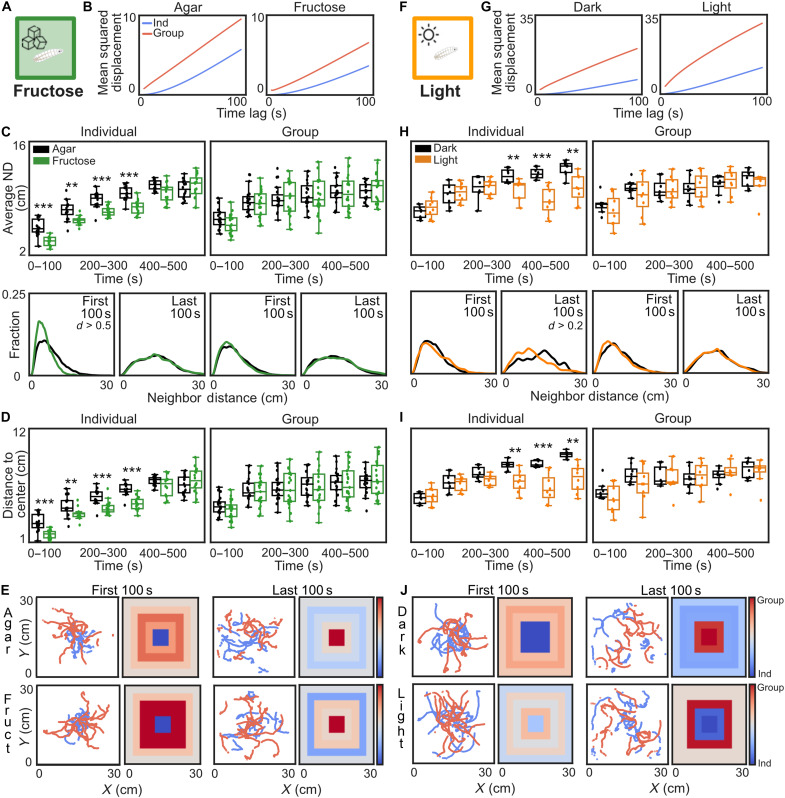
Social context modulation overrides responses to several external stimuli. (**A**) Larvae were tested on a 2 M fructose/agar substrate. (**B**) MSD for each treatment, comparing superimposed groups (*N* = 16 per treatment; blue) and real groups (*N* = 20 per treatment; red). (**C**) Top: AND over time for the superimposed and real groups. Bottom: ND for the first and last 100 s of the 10-min experiment. Left: superimposed groups, Right: real groups (Effect size = Cohen’s *d* (0.2 < *d* < 0.5 - small; 0.5 < *d* < 0.8 - moderate). black = agar, green = fructose. (**D**) Average distance from the center over time for superimposed and real groups. (**E**) Sample trajectories and distribution heatmaps (superimposed groups = blue, real groups = red). (**F**) Larvae were tested in an arena with white light LEDs at the arena border. (**G**) MSD for each treatment, comparing superimposed groups (*N* = 8 per treatment, blue) and real groups (*N* = 8 per treatment, red). (**H**) Top: AND over time for the superimposed and real groups. Bottom: ND for the first and last 100 s of the 10-min experiment. Left: Superimposed groups. Right: Real groups. Black = light off; orange = light on. (**I**) Average distance from the center over time for superimposed and real groups. (**J**) Sample trajectories and distribution heatmaps (superimposed groups = blue; real groups = red). (Bootstrapped CI test for individuals and Mann-Whitney *U* test for groups = **P < 0.01, and ***P < 0.001).

First, larvae were tested on an evenly distributed 2 M fructose/agar substrate, which is usually attractive (Fig. 2A) (*30*). The social modulation was reproducible on all substrates; larvae in the group dispersed more than individual larvae (Fig. 2B). AND, ND, and distance to center suggest that individuals stay in the middle and have reduced dispersal on fructose (Fig. 2, C and D). Individuals seem to dwell in the center due to fructose attraction; real groups, however, behave the same on both substrates (Fig. 2, D and E).

Second, larvae were tested on a pure agar substrate illuminated with white light-emitting diodes (LEDs) from the arena borders, a stimulus they usually avoid (Fig. 2F) (*31*). The social context modulation was reproducible in the light and dark arena; larvae in the group dispersed more than individual larvae (Fig. 2G). In the first half of the light-on experiment, individual larvae showed high dispersal, potentially due to light exposure arousal (Fig. 2, H to J). In the second half of the experiment, they even seemed to avoid the bright borders with the LEDs and moved toward the arena center, resulting in a low AND and distance to center (Fig. 2, H to J). However, no such light avoidance response was visible in the real group (Fig. 2, H to J). These results suggest that social context modulation can override responses to nonfood and nonoptimal food cues.

### Developmental experience influences social modulation

**Fig. 3.**
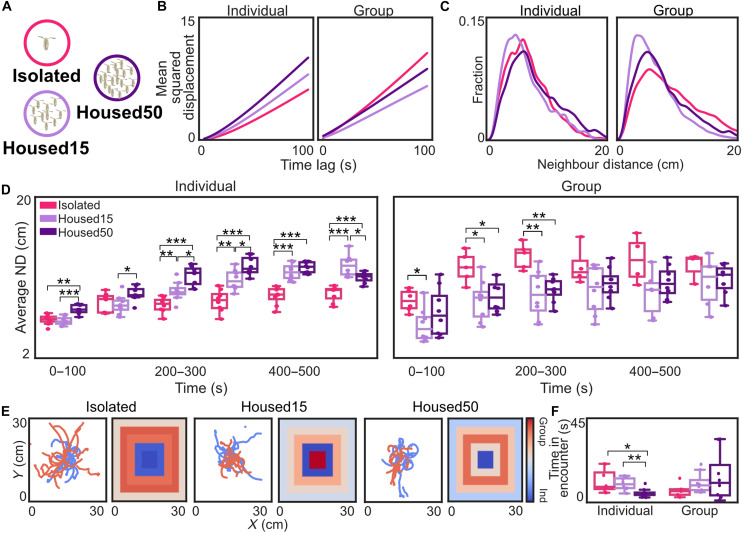
Developmental experience influences social modulation. (**A**) Larvae were reared under three different conditions: isolated from a single egg (pink), in small groups of 15 eggs per well (light purple), or crowded conditions with 50 eggs per well (dark purple). (**B**) MSD was plotted for each treatment for the superimposed groups (*N* = 8 per treatment) and real groups (*N* = 5 for isolation; *N* = 8 for Housed15/50). (**C**) ND for the first 100 s. (**D**) AND over time for the superimposed and real groups. Statistical comparison was performed between pairs of each of the treatments, which were all measured in parallel. (**E**) Sample trajectories and distribution heatmaps. (**F**) Time in encounter for the real and superimposed groups over the first 100 s. (Bootstrapped CI test for individuals and Mann-Whitney *U* test for groups = *P < 0.05, **P < 0.01, and ***P < 0.001).

We next asked whether social context modulation is an innate behavioral response or whether it is affected by prior conspecific exposure. Therefore, we reared larvae under isolated, mildly crowded, and highly crowded conditions (Fig. 3A).

For isolation, we placed individual eggs in separate wells (48-well plates) filled with fly food, allowing them to develop without any conspecific interaction throughout development. For mildly and highly crowded conditions, we placed 15 or 50 fly eggs in a single well (48-well plate) filled with fly food, respectively. We observed unhatched eggs under all conditions, probably due to damage from egg handling. Under the isolated condition, we observed further decrease in survival probably because larvae could not process the food alone. The treatments led to differences in larval size, but locomotion was unaffected in individually tested larvae (fig. S2, A and C).

When tested individually, isolated larvae and larvae from the mildly crowded condition dispersed slowly away from the center and had lower AND and ND than larvae from the highly crowded condition (Fig. 3, B to E, and fig. S2D). When tested in groups, larvae from the isolated condition dispersed even more than larvae from the two crowded conditions and had higher AND and ND. Crowded larvae tended to spend more time in encounters with conspecifics in real groups compared to isolated larvae (Fig. 3F). These findings suggest that developmental social experience affects larval behavior, where isolation leads to increased conspecific avoidance.

### Social context modulation requires the detection of multimodal sensory cues

**Fig. 4.**
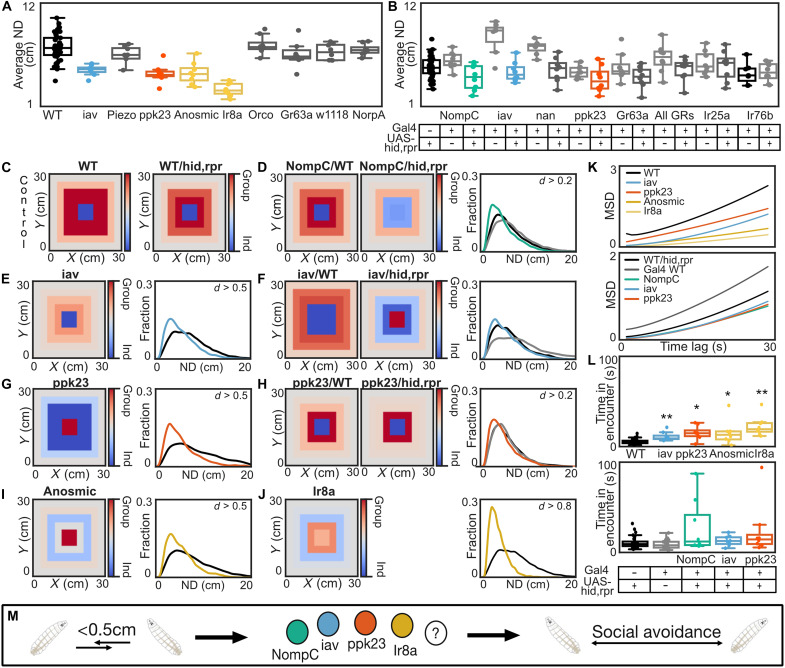
In real groups, conspecifics are recognized by multimodal sensory cues. (**A**) Mutant experiments: Real group AND for the first 100 s (see fig. S3A for statistical comparisons). WT results are pooled over all experiments (black; *N* = 54). Mutant lines with no phenotype (gray; *N* = 8) and mutant hits (different colors; *N* = 8). (**B**) Genetic silencing: Real group average ND for the first 100 s (see fig. S3B for statistical comparisons). *WTx hid-reaper* (indicated by hid,rpr) control results are pooled over all experiments (black; *N* = 40). Experimental crosses/GAL4 controls with no phenotype (gray; *N* = 8). Experimental cross hits (different colors; *N* = 8). (**C**) Distribution heatmaps for the pooled controls: WT (*N* = 24) and *WT x hid/reaper* (*N* = 24). (**D** to **J**) Distribution heatmaps [superimposed groups (blue) and real groups (red)]. ND for the first 100 s in all real groups. Effect size = Cohen’s *d* (0.2 < *d* < 0.5: small; 0.5 < *d* < 0.8: moderate; *d* > 0.8: high). Controls = black/gray; mutants/experimental groups = different colors. (**K**) MSD for all hits/pooled controls. (**L**) Time spent in encounters for all hits. (**M**) Social context modulation is mediated via four different mechano- and chemosensory channels and receptors.

Larval behavior is modulated by social context; thus, we asked how larvae sense conspecifics. We leveraged the genetic toolkit available in *Drosophila* to test sensory receptor mutants (Fig. 4A) and larvae with genetically ablated sensory neurons (Fig. 4B and table S1).

To ablate sensory neurons, we crossed the sensory neuron GAL4-driver lines with *UAS-hid*,*reaper* (*32*) to induce cell-specific apoptosis (*33*). Individual larvae were tested in parallel to control for locomotion deficits or other behavioral changes (figs. S3, A and B, S4, A to J, and S5, A to G). We identified that different sensory modality impairments affect social context modulation in real group experiments (Fig. 4, A and B).

Larvae with impaired mechanosensation (inactive = *iav* mutant; NompC = *NompC x hid*,*reaper*) show reduced AND. The mechanosensitive channels iav and NompC have been shown to detect the touch and vibration of conspecifics in adults (*15*, *23*). Knockout of the mechanosensitive piezo channel (mutant) or ablation of nanchung (nan) expressing neurons (*nan x hid*,*reaper*) did not affect social dispersal (Fig. 4, A and B). Impairing chemosensation (ppk23 = *ppk23 x hid*,*reaper* and *ppk23* mutant; Ionotropic receptor 8a = *IR8a* mutant) also induces reduced AND. Anosmic mutants (*23*), which have mutations in *orco*, *Ir8a*, *IR25a*, and *GR63a*, show a similar reduction in the AND as *IR8a* mutants (*23*). As we find no impairment in social distancing in the *orco* mutant, *GR63a* mutant, and with *IR25a x hid*,*reaper* (see also figs. S3, A and B, and S7, A to K), we conclude that the anosmic phenotype is due to the *IR8a* mutation. Testing a novel *IR8a* mutant (Ir8a′) confirmed the phenotype (fig. S6) (*34*). We also used the GAL4/UAS system to silence IR8a-positive cells; however, we could not detect a phenotype. This is in line with published results that indicate no detectable expression in both GAL4 lines (*35*). ppk23 is a receptor involved in sensing larval deposits and egg pheromones (*27*, *28*). We find that it is also required for normal social dispersal behavior in fly larvae. The w1118 control, blind *NorpA* mutants in dark or in light (fig. S7L) (*36*), and silencing of all gustatory receptors (all GRs = *all GRs x hid*,*reaper*) (*37*) or IR76b expressing neurons (*IR76b x hid*,*reaper*) (*35*) showed no difference to WT behavior in real groups (fig. S7, A to K). The all GR-GAL4 line has been recently published and labels many GR-positive neurons in the larval head; ablation of these neurons impaired taste responses and agar hardness preferences (*37*).

Real group ANDs for the WT control and the *WT x hid*,*reaper* control did not differ across experiments, and thus, Fig. 4 (A and B) includes a pooled dataset (fig. S3, A and B). We also visualized the average positional distribution of the WT control and the *WT x hid*,*reaper* control in a single heatmap plot (Fig. 4C). All hits have a low ND and spread out less than individuals (Fig. 4, D to J). The *ppk23* receptor mutant phenotype was stronger than the genetic ablation experiment; this could be explained by an incomplete GAL4 expression pattern or weak GAL4 expression by the driver line. Across all hits, larvae in real groups had a lower dispersal rate (Fig. 4K), stayed closer to the center (fig. S4, I and J), and spent more time in encounters in the first 100 s (Fig. 4L), indicating that they stay closer together. This is, however, not due to locomotion defects, as individually tested larvae show almost no difference in ND nor dispersal in the arena (figs. S4, A to J, and S5, A to G). In summary, we find that socially induced dispersal in fly larvae requires multimodal sensing via touch, ppk23, and Ir8a receptors (Fig. 4M).

## DISCUSSION

We find that conspecific exposure affects *Drosophila* larval behavior in a nonfood context. Larvae in a group show higher dispersal than individual larvae in the arena. The effect of social context on larval behavior is robust and prioritized over several other external cues, such as aversive light and attractive sugar. Isolated larvae show an enhanced dispersal in the presence of conspecifics; thus, larvae adapt to their social environment during development. Last, we show that conspecifics provide multimodal sensory cues and are recognized through mechanosensory and chemosensory pathways.

### Dispersal in the group context

In a nonfood context, larvae usually explore an experimental arena as they search for food. We show that individual larvae show slow exploration and perform local search patterns; they stop more often and make bigger turns (fig. S1, A and B). However, when tested in a group, larvae disperse faster and perform straight, fast runs away from conspecifics after an encounter (fig. S1H) (*26*). Encounters with alive conspecifics seem required to induce social modulation, as adding agar piece obstacles into the arena does not enhance dispersal in individual larvae (movie S5 and fig. S1J). Most individual larvae are retained in the center by the obstacles, but larvae in the group still disperse away from the center, probably due to social modulation. Most conspecific encounters take place in the first 100 s of the experiment (Fig. 1E); however, larval dispersal behavior is modulated over the whole 10-min experiment (MSD; Fig. 1C). These findings suggest that the initial exposure to conspecifics induces a shift in the internal state that lasts over many minutes. Social modulation might be beneficial in the absence of food, as larvae compete for potential food sources and might themselves become a food source for conspecifics (*22*). Social avoidance during exploration might also allow for faster detection of any food source in the environment by a group of larvae. In the presence of protein-rich fly food, larval dispersal is reduced, and we find no social modulation (movie S4 and fig. S1F). This is in line with previous studies, which show that groups of larvae even perform collective digging in fly food (*19*).

### Social cues versus external cues

We tested individuals and groups of larvae in different sensory contexts and found that individual larvae responded stronger to an attractive fructose substrate or an aversive light source (Fig. 2). Individual larvae dispersed less on fructose, probably due to immediate exploitation of the sweet and nutritious fructose substrate. Grouped larvae dispersed similarly on both substrates, and thus, social context overwrote the response to fructose. Fructose is sweet and attractive; however, it is not an optimal food source for larvae, as they require protein for development (*38*). In addition, individual larvae dispersed faster when we illuminated the arena with white LED lights. We hypothesize that this is due to the stress caused by aversive light (*31*). As the LED lights were placed at the arena borders, they created a weak light gradient toward the middle of the arena. Therefore, the individual larvae returned to the middle of the arena, as they were avoiding the illuminated borders. However, grouped larvae dispersed similarly in the illuminated or dark arena, and thus, social context also overwrote the response to light over the whole time of the experiment. These results show that social context can override responses to other sensory inputs and is a strong modulator of larval behavior. We, however, also find that stronger external stimuli, such as protein-rich food and attractive agar obstacles (movie S4 and fig. S1), partially or fully override the social modulation effect of the conspecifics. Exposure to agar obstacles could, however, also limit interactions between larvae and, thus, reduce the social modulation effect in the experiment. This might also be possible with other highly salient stimuli, such as pure odors (*39*, *40*) or on/off light choices (*31*). Future experiments will reveal how social context is integrated with other sensory cues and how it can suppress certain behavioral responses.

The social modulation effects are highly reproducible in our big arena (25 cm by 25 cm). Many other behavioral larval group assays use a smaller arena size (⌀ 10 cm), restricting the exploration range. Although the social modulation effect might be less obvious when testing groups or individuals in these smaller assays, conspecifics might still influence larval responses to weak external cues.

### Social experience

We tested whether social context modulation is an innate behavior or whether it is shaped during development. Testing isolated larvae, which had never encountered another larva before, in a group revealed an increased social modulation effect (Fig. 3). Thus, during normal development, larvae often encounter conspecifics in the presence of food and might thus be less aroused by conspecific exposure. Flies can learn about the conspecific presence and show decreased stress responses afterward (*24*); the same might be true for larvae (*41*). Larvae from the two differently crowded conditions dispersed less than isolated larvae and stayed closer together when tested in a group. Rearing larvae under highly crowded conditions led to fast dispersal in individually tested animals, which might be due to competition, stress, or reduced food access. Investigating how food deprivation affects individual and grouped larval behavior will require further examination.

In general, animals exhibit plasticity in their sensory processing and behavioral decisions in response to isolation or conspecifics (*42*–*46*). Thus, uncovering how neural circuits adapt to social experiences in *Drosophila* larvae might also reveal general principles and mechanisms that can be adapted to other species. In fly larvae, combining experience-dependent plasticity with high-throughput connectomic studies is feasible, dissectable, and one of the next frontiers in neuroscience.

### Conspecifics are recognized via multimodal sensory cues

Our results suggest that encounters with conspecifics seem to be the main driving force of social modulation and the induction of a social state. Therefore, we looked at how larvae sense conspecific presence. We find that mechanosensory (NompC and iav) and chemosensory (ppk23 and IR8a) receptors are required for social modulation. The mechanosensory neurons expressing NompC and iav are required for sensing gentle touch and vibrations in adult flies and larvae (*15*, *19*). Thus, fly larvae are probably capable of sensing the touch or vibrations of other live larvae. ppk23 is known to be required for larval deposit preference and mediates repulsion to fly eggs, preventing egg cannibalism (*27*, *28*). In adult flies, ppk23 is involved in courtship behavior (*47*). We find that ppk23 is necessary for social dispersal in larvae, supporting its role in mediating aversion in an alive larva context (*28*). We hypothesize that ppk23 may generally be required for species recognition but in a context-dependent manner. It may drive aversion in the presence of live conspecifics when no food is available (*28*) while promoting attraction when food or attractive cues are present but no other alive conspecifics (*27*). Last, we observe a strong phenotype in *IR8a/IR8a′/*anosmic mutants using two different mutations in the *IR8a* gene. While previous literature suggests no known IR8a expression in larvae, sensitive polymerase chain reaction experiments indicate the presence of *IR8a* mRNA (*35*, *48*, *49*). Testing two different IR8a-GAL4 lines, we could not detect any phenotype in our assay, which is in line with a lack of green fluorescent protein detection when using this driver line (*35*). IR8a is required for acid sensing in adult flies and mosquitoes (*50*–*53*). Further investigation is needed to elucidate the potential role of Ir8a in mediating social interactions in larvae.

We cannot exclude that additional receptors or sensors might be involved in conspecific recognition; we might not have found a phenotype in our screening due to redundant requirements. In future experiments, silencing multiple receptors might reveal such additional sensors. In summary, larval conspecifics are recognized via multimodal sensory pathways, similar to what was found in adult flies (*15*). Uncovering the sensory systems required for social context–dependent modulation paves the way for dissecting the central brain circuits involved in conspecific recognition, and understanding how social cues are integrated with and can overwrite other sensory processing pathways.

Animals make complex behavioral decisions based on their internal state (*54*), experience (*24*), sensory context (*42*, *55*, *56*), and social context (*23*). We show that conspecifics also affect larval *Drosophila* behavior in a nonfood context and the presence of environmental cues. These findings highlight the general importance of social exposure during development and of the social influence in group experiments.

Social cues are important in our daily lives, and social isolation can severely affect human health (*7*). *Drosophila* shares homologous genes and conserved neural subtypes with all animals. The available genetic toolkit and the whole-brain connectome in *Drosophila* offer a distinct advantage over other taxa, as they enable precise dissection of the neural circuits underlying social interactions. This will facilitate the investigation of the neural basis of social behavior and health in other taxa ranging from insects to mammals.

## MATERIALS AND METHODS

### Animal stocks and husbandry

Flies were raised on a standard cornmeal diet and kept in incubators at 25°C and 65% humidity on a 12-hour light/dark cycle. Adult flies were allowed to lay eggs for 48 hours and were then removed from the vials. The eggs were allowed to develop for 4 to 6 days. Larvae in the middle to late second instar stage were used for experimentation.

### Transgenic lines

Mutant fly lines and GAL4/UAS crosses for experiments were maintained as mentioned above and crossed with a specific number of animals to maintain similar crowding during development. Mutant lines were not backcrossed to WT background, but individual larva experiments and validation with multiple genetic lines control for genetic background effects. For a single cross, at least 10 GAL4-driver line males and 25 UAS-hid/reaper female virgins were collected and allowed to mate. Control crosses for both the UAS-hid/reaper and the GAL4-driver line crossed to WT were also made with a similar sex ratio. WT experiments were performed with the Canton-S strain. Stocks were obtained from the Bloomington *Drosophila* Stock Center (BDSC) or courtesy of other labs (see table S1).

### Behavioral experiments

#### Social modulation

Either a single larva or a group of 15 larvae was allowed to roam freely on a 25 cm–by–25 cm assay plate filled with 100 ml of a 2% agarose substrate. The arenas were maintained at a constant temperature of 25°C and 60% humidity and placed in a light-tight box. Larval behavior was recorded for 10 min with a Basler camera (acA2040-90umNIR) and lens (Kowa Lens LM16HC F1.4 f15mm1") at 1 frame per second with a red light filter (Edmund Optics, #89-837) above the arena. The arena was illuminated with red light using infrared LEDs from the bottom (SOLAROX LED strip infrared 940 nm). Movies S1, S3, S4, and S5 were recorded in a smaller arena (diameter: 13 cm) for only 100 s and higher spatial resolution to visualize larval behavior.

For all behavioral experiments, middle to late second-stage instar larvae of a similar size (4 to 6 days after egg laying) were used. Larvae were removed from the fly vials and washed in distilled water to remove all traces of food. To study individual behavior, a single larva was placed in the middle of the assay plate. These experiments were repeated with different larvae at least 30 times per experimental condition. To study group behavior, 15 larvae, if not stated otherwise, were placed in the middle of the agarose plate. These experiments were repeated at least eight times, if not stated otherwise.

When testing larval behavior in the presence of fly food, a small piece of food (1 to 2 g of standard cornmeal food) was placed in the center of the arena. Fifteen larvae were placed in the center, and this was repeated eight times.

To test whether physical encounters are sufficient to enhance dispersal, we placed 14 obstacles of 2% agar (3 to 4 mm) in the center of the arena. Larvae were placed in the center of the obstacles. Individual larvae experiments were repeated 30 times, and 15 larvae experiments were repeated eight times.

#### Change in arena context

To test whether an attractive substrate influences social modulation, larvae were tested on a substrate containing fructose. Therefore, we mixed agarose with D-(-)-Fructose (Merck, CAS #57-48-7) and poured it into the square arena plates (for one plate: 36 g/100 ml = 2 M).

To test whether the light environment influences social modulation, larvae were tested in an illuminated arena. Therefore, we placed strips of white light LEDs (SOLAROX 12 V LED strip reel warm white) on three sides of the experimental box, which illuminated the agarose plate from the side. One side was not illuminated, as this was the side where the arena was closed with a black curtain. No specific side preference was detected in the larvae.

#### Isolation assay

Flies were allowed to lay eggs for 24 hours on standard fly food in a fly cage. The eggs were collected the next day and placed in separate wells containing standard fly food in a 48-well plate. For the isolation treatment, a single egg was placed in one well. Each well was closed with a cotton plug to prevent larval interactions. The food and cotton plug were sprayed with water to prevent the food from drying out. For the housed 15 and housed 50 treatments, 15 and 50 eggs were placed in a single well, respectively. All plates were maintained at a constant temperature of 25°C and humidity of 65%. After 4 to 6 days, the larvae were removed from the well plates and tested using the assay setup as described before.

After egg collection, 65% of the housed 15 larvae could be used for experiments. From the isolated eggs, 35% of larvae could be used for experiments. Reduced survival is due to egg handling under both conditions. Monitoring larval survival during isolation treatment, we observed that 72% of larvae hatched from the handled eggs a day after isolation. A further decrease in survival during development (35%) is very likely due to individual larvae not being able to properly process the food alone.

### Behavioral analysis

Videos were analyzed using the freely available tracking software TRex (*29*) to obtain the position (*X*/*Y*), speed, bending angle, and size of all tested larvae (data S1 and S2 and movie S2). Tracking of larvae using TRex was analyzed to make sure that there was 98% accuracy throughout the experiment.

To quantify larval dispersal, we calculated the MSD for specific time lags (τ)

$$\text{MSD}\;(\tau )={\left[x(t+\tau )-x(t)\right]}^{2}+{\left[y(t+\tau )-y(t)\right]}^{2}$$

At each frame, the MSD has been calculated for all the past τ frames, if possible. For each τ, the average MSD has been calculated as the mean of all the MSD values for that τ. We then calculated the MSD for each treatment as the average MSD of all larvae across all trials. The slope of the linear fit to MSD curve provides insights into the diffusion properties of the larval groups (data S3). We additionally calculated the distance from the center over time to plot the difference from their starting position.

To visualize the position of the larvae, we plotted their location in a probability heatmap. The heatmap was made by subtracting the positional data of the average of all artificial groups from the average of all real groups. After subtracting, the values of each region were averaged out in concentric squares to focus on the distance from the center, the initial position of the larva. A positive value indicates a higher probability of finding a larva from a group at this location (red), and a negative value indicates the more likely presence of individuals (blue).

On the basis of *X*/*Y* coordinates, the AND of each larva was calculated to all its neighbors in the real group. We compared the AND of eight real groups to the AND of eight superimposed groups, which were generated from a subset of the individually tested larvae. We picked 15 unique individual tracks from a pool of at least 30 individually tested larvae for one superimposed group. We have done bootstrapping for a subset of the experiments with an independent dataset and compared it to the bootstrapping of a dependent dataset (fig. S8) to ensure that the repeated sampling is not affecting the results being observed. The ND was also calculated between each pair of larvae within a real or superimposed group and plotted as a kernel density estimate plot to visualize the distribution of the distances between the pairs.

To quantify local interactions, we counted time (1 frame = 1 s) in larval encounters, which were defined by the number of frames in which a larval pair was less than 0.5 cm apart. Encounter duration was calculated by adding consecutive frames within a single encounter (seconds per encounter). Run speed was defined as the average speed of the larva when moving between 0.5 and 2 mm/s. The percentage of stops was calculated as the proportion of time when the speed was below 0.5 mm/s during this period. To analyze turn behavior, we plotted the average bending angle, and the percentage of small turns and big turns was calculated as the number of values where the bending angle was less than 0.4 rad and more than 0.4 rad, respectively. The speed and bending angle of larvae were also specifically calculated during and after (5 s) the encounter. Box plots or density plots are shown to visualize the data. Box plots indicate the first and third quartiles with the median. Whiskers indicate the 5 and 95 percentiles of the data. Dots indicate group averages from individual experiments.

### Statistical analysis

To detect significant differences between experimental groups (ND and time in encounter), we performed a nonparametric Mann-Whitney *U* test, which can be used even for not normally distributed data (Mann-Whitney *U* test = *P < 0.05, **P < 0.01, and ***P < 0.001). The test was conducted with treatment (social context, environment, or genotype) as the grouping factor. For percentages (stops, small turns, and big turns), the data were converted to a normal distribution using arcsine. For bootstrapped individual data, we performed a bootstrapped comparison between medians for a more conservative statistical estimate. To compare ND across groups, we calculated the Cohen’s *d* effect sizes to quantify the magnitude of differences between the two groups. The values of effect size were interpreted as high (*d* > 0.8), moderate (0.5 < *d* < 0.8), small (0.2 < *d* < 0.5), and negligible (*d* < 0.2).

## References

[1] A. J. King, G. Cowlishaw, When to use social information: The advantage of large group size in individual decision making. Biol. Lett. 3, 137–139 (2007). 17284400 10.1098/rsbl.2007.0017 PMC2104485

[2] N. Miller, S. Garnier, A. T. Hartnett, I. D. Couzin, Both information and social cohesion determine collective decisions in animal groups. Proc. Natl. Acad. Sci. U.S.A. 110, 5263–5268 (2013). 23440218 10.1073/pnas.1217513110 PMC3612658

[3] M. A. Gil, Z. Emberts, H. Jones, C. M. S. Mary, Social information on fear and food drives animal grouping and fitness. Am. Nat. 189, 227–241 (2017). 28221835 10.1086/690055

[4] C. C. Ioannou, V. Guttal, I. D. Couzin, Predatory fish select for coordinated collective motion in virtual prey. Science 337, 1212–1215 (2012). 22903520 10.1126/science.1218919

[5] M. Andersson, Y. Iwasa, Sexual selection. Trends Ecol. Evol. 11, 53–58 (1996). 21237761 10.1016/0169-5347(96)81042-1

[6] S. Bazazi, C. Buhl, J. J. Hale, M. L. Anstey, G. A. Sword, S. J. Simpson, I. D. Couzin, Collective motion and cannibalism in locust migratory bands. Curr. Biol. 18, 735–739 (2008). 18472424 10.1016/j.cub.2008.04.035

[7] D. Bzdok, R. I. M. Dunbar, Social isolation and the brain in the pandemic era. Nat. Hum. Behav. 6, 1333–1343 (2022). 36258130 10.1038/s41562-022-01453-0

[8] S. J. Simpson, E. Despland, B. F. Hägele, T. Dodgson, Gregarious behavior in desert locusts is evoked by touching their back legs. Proc. Natl. Acad. Sci. U.S.A. 98, 3895–3897 (2001). 11274411 10.1073/pnas.071527998 PMC31149

[9] S. M. Rogers, T. Matheson, E. Despland, T. Dodgson, M. Burrows, S. J. Simpson, Mechanosensory-induced behavioural gregarization in the desert *locustSchistocerca gregaria*. J. Exp. Biol. 206, 3991–4002 (2003). 14555739 10.1242/jeb.00648

[10] S. Gupta, A. Cribellier, S. B. Poda, O. Roux, F. T. Muijres, J. A. Riffell, Mosquitoes integrate visual and acoustic cues to mediate conspecific interactions in swarms. Curr. Biol. 34, 4091–4103.e4 (2024). 39216484 10.1016/j.cub.2024.07.043 PMC11491102

[11] B. Hölldobler, Multimodal signals in ant communication. J. Comp. Physiol. 184, 129–141 (1999).

[12] R. Harpaz, M. N. Nguyen, A. Bahl, F. Engert, Precise visuomotor transformations underlying collective behavior in larval zebrafish. Nat. Commun. 12, 6578 (2021). 34772934 10.1038/s41467-021-26748-0 PMC8590009

[13] C. Dulac, Sensory coding of pheromone signals in mammals. Curr. Opin. Neurobiol. 10, 511–518 (2000). 10981622 10.1016/s0959-4388(00)00121-5

[14] A. F. Simon, M.-T. Chou, E. D. Salazar, T. Nicholson, N. Saini, S. Metchev, D. E. Krantz, A simple assay to study social behavior in *Drosophila*: Measurement of social space within a group^1^. Genes Brain Behav. 11, 243–252 (2012). 22010812 10.1111/j.1601-183X.2011.00740.x PMC3268943

[15] L. Jiang, Y. Cheng, S. Gao, Y. Zhong, C. Ma, T. Wang, Y. Zhu, Emergence of social cluster by collective pairwise encounters in *Drosophila*. eLife 9, e51921 (2020). 31959283 10.7554/eLife.51921 PMC6989122

[16] J. Schneider, M. H. Dickinson, J. D. Levine, Social structures depend on innate determinants and chemosensory processing in *Drosophila*. Proc. Natl. Acad. Sci. U.S.A. 109, 17174–17179 (2012). 22802679 10.1073/pnas.1121252109 PMC3477376

[17] B. Shorrocks, *Drosophila: Invertebrate Types* (Ginn, 1972).

[18] T. A. Markow, The secret lives of *Drosophila* flies. eLife 4, e06793 (2015). 26041333 10.7554/eLife.06793 PMC4454838

[19] M. Dombrovski, L. Poussard, K. Moalem, L. Kmecova, N. Hogan, E. Schott, A. Vaccari, S. Acton, B. Condron, Cooperative behavior emerges among *Drosophila* larvae. Curr. Biol. 27, 2821–2826.e2 (2017). 28918946 10.1016/j.cub.2017.07.054

[20] Z. Durisko, R. Kemp, R. Mubasher, R. Dukas, Dynamics of social behavior in fruit fly larvae. PLoS ONE 9, e95495 (2014). 24740198 10.1371/journal.pone.0095495 PMC3989340

[21] M. Trienens, M. Rohlfs, A potential collective defense of *Drosophila* larvae against the invasion of a harmful fungus. Front. Ecol. Evol. 8, 79 (2020).

[22] R. K. Vijendravarma, S. Narasimha, T. J. Kawecki, Predatory cannibalism in *Drosophila melanogaster* larvae. Nat. Commun. 4, 1789 (2013). 23653201 10.1038/ncomms2744

[23] P. Ramdya, P. Lichocki, S. Cruchet, L. Frisch, W. Tse, D. Floreano, R. Benton, Mechanosensory interactions drive collective behaviour in *Drosophila*. Nature 519, 233–236 (2015). 25533959 10.1038/nature14024 PMC4359906

[24] V. Lobato-Rios, T. K. C. Lam, P. Ramdya, Conspecific sociability is regulated by associative learning circuits. bioRxiv 2024.11.25.624845 [Preprint] (2024). 10.1101/2024.11.25.624845.

[25] E. D. Justice, N. J. Macedonia, C. Hamilton, B. Condron, The simple fly larval visual system can process complex images. Nat. Commun. 3, 1156 (2012). 23093193 10.1038/ncomms2174

[26] N. Otto, B. Risse, D. Berh, J. Bittern, X. Jiang, C. Klämbt, Interactions among *Drosophila* larvae before and during collision. Sci. Rep. 6, 31564 (2016). 27511760 10.1038/srep31564 PMC4980675

[27] J. D. Mast, C. M. D. Moraes, H. T. Alborn, L. D. Lavis, D. L. Stern, Evolved differences in larval social behavior mediated by novel pheromones. eLife 3, e04205 (2014). 25497433 10.7554/eLife.04205 PMC4270068

[28] S. Narasimha, K. O. Nagornov, L. Menin, A. Mucciolo, A. Rohwedder, B. M. Humbel, M. Stevens, A. S. Thum, Y. O. Tsybin, R. K. Vijendravarma, *Drosophila melanogaster* cloak their eggs with pheromones, which prevents cannibalism. PLOS Biol. 17, e2006012 (2019). 30629594 10.1371/journal.pbio.2006012 PMC6328083

[29] T. Walter, I. D. Couzin, TRex, a fast multi-animal tracking system with markerless identification, and 2D estimation of posture and visual fields. eLife 10, e64000 (2021). 33634789 10.7554/eLife.64000 PMC8096434

[30] A. Schipanski, A. Yarali, T. Niewalda, B. Gerber, Behavioral analyses of sugar processing in choice, feeding, and learning in larval *Drosophila*. Chem. Sens. 33, 563–573 (2008). 10.1093/chemse/bjn024 PMC2467463 18511478

[31] E. P. Sawin-McCormack, M. B. Sokolowski, A. R. Campos, Characterization and genetic analysis of *Drosophila Melanogaster* photobehavior during larval development. J. Neurogenet. 10, 119–135 (1995). 8592272 10.3109/01677069509083459

[32] A. Huser, A. Rohwedder, A. A. Apostolopoulou, A. Widmann, J. E. Pfitzenmaier, E. M. Maiolo, M. Selcho, D. Pauls, A. Von Essen, T. Gupta, S. G. Sprecher, S. Birman, T. Riemensperger, R. F. Stocker, A. S. Thum, The serotonergic central nervous system of the *Drosophila* larva: Anatomy and behavioral function. PLoS ONE 7, e47518 (2012). 23082175 10.1371/journal.pone.0047518 PMC3474743

[33] K. White, E. Tahaoglu, H. Steller, Cell killing by *the Drosophila* gene *reaper*. Science 271, 805–807 (1996). 8628996 10.1126/science.271.5250.805

[34] O. Kanca, J. Zirin, J. Garcia-Marques, S. M. Knight, D. Yang-Zhou, G. Amador, H. Chung, Z. Zuo, L. Ma, Y. He, W.-W. Lin, Y. Fang, M. Ge, S. Yamamoto, K. L. Schulze, Y. Hu, A. C. Spradling, S. E. Mohr, N. Perrimon, H. J. Bellen, An efficient CRISPR-based strategy to insert small and large fragments of DNA using short homology arms. eLife 8, e51539 (2019). 31674908 10.7554/eLife.51539 PMC6855806

[35] V. Croset, M. Schleyer, J. R. Arguello, B. Gerber, R. Benton, A molecular and neuronal basis for amino acid sensing in the *Drosophila* larva. Sci. Rep. 6, 34871 (2016). 27982028 10.1038/srep34871 PMC5159833

[36] B. T. Bloomquist, R. D. Shortridge, S. Schneuwly, M. Perdew, C. Montell, H. Steller, G. Rubin, W. L. Pak, Isolation of a putative phospholipase c gene of *drosophila*, norpA, and its role in phototransduction. Cell 54, 723–733 (1988). 2457447 10.1016/s0092-8674(88)80017-5

[37] N. Komarov, C. Fritsch, G. L. Maier, J. Bues, M. Biočanin, C. B. Avalos, A. Dodero, J. Y. Kwon, B. Deplancke, S. G. Sprecher, Food hardness preference reveals multisensory contributions of fly larval gustatory organs in behaviour and physiology. PLOS Biol. 23, e3002730 (2025). 39883595 10.1371/journal.pbio.3002730 PMC11781724

[38] S. Schwarz, Z. Durisko, R. Dukas, Food selection in larval fruit flies: Dynamics and effects on larval development. Naturwissenschaften 101, 61–68 (2014). 24352256 10.1007/s00114-013-1129-z

[39] B. Gerber, T. Hendel, Outcome expectations drive learned behaviour in larval *Drosophila*. Proc. R. Soc. B Biol. Sci. 273, 2965–2968 (2006). 10.1098/rspb.2006.3673 PMC1639518 17015355

[40] T. Niewalda, I. Jeske, B. Michels, B. Gerber, ‘Peer pressure’ in larval *Drosophila* ? Biol. Open 3, 575–582 (2014). 24907371 10.1242/bio.20148458 PMC4154293

[41] Z. Slepian, K. Sundby, S. Glier, J. McDaniels, T. Nystrom, S. Mukherjee, S. T. Acton, B. Condron, Visual attraction in *Drosophila* larvae develops during a critical period and is modulated by crowding conditions. J. Comp. Physiol. A 201, 1019–1027 (2015). 10.1007/s00359-015-1034-3 26265464

[42] C. M. Jernigan, F. M. Uy, Impact of the social environment in insect sensory systems. Curr. Opin. Insect Sci. 59, 101083 (2023). 37423425 10.1016/j.cois.2023.101083

[43] R. Harpaz, M. Phillips, R. Goel, M. C. Fishman, F. Engert, Experience-dependent modulation of collective behavior in larval zebrafish. bioRxiv 2024.08.02.606403[Preprint] (2024). 10.1101/2024.08.02.606403. PMC13451335 42362575

[44] H. F. Harlow, R. O. Dodsworth, M. K. Harlow, Total social isolation in monkeys. Proc. Natl. Acad. Sci. U.S.A. 54, 90–97 (1965). 4955132 10.1073/pnas.54.1.90 PMC285801

[45] M. Toth, E. Mikics, A. Tulogdi, M. Aliczki, J. Haller, Post-weaning social isolation induces abnormal forms of aggression in conjunction with increased glucocorticoid and autonomic stress responses. Horm. Behav. 60, 28–36 (2011). 21316368 10.1016/j.yhbeh.2011.02.003

[46] A. H. Groneberg, J. C. Marques, A. L. Martins, R. Diez Del Corral, G. G. De Polavieja, M. B. Orger, Early-life social experience shapes social avoidance reactions in larval zebrafish. Curr. Biol. 30, 4009–4021.e4 (2020). 32888479 10.1016/j.cub.2020.07.088

[47] B. Lu, A. LaMora, Y. Sun, M. J. Welsh, Y. Ben-Shahar, ppk23-dependent chemosensory functions contribute to courtship behavior in *Drosophila melanogaster*. PLOS Genet. 8, e1002587 (2012). 22438833 10.1371/journal.pgen.1002587 PMC3305452

[48] M. Z. Ali, A. Anushree, A. L. Bilgrami, A. Ahsan, M. S. Ola, R. Haque, J. Ahsan, Phenylacetaldehyde induced olfactory conditioning in *Drosophila melanogaster* (Diptera: Drosophilidae) larvae. J. Insect Sci. 23, 25 (2023). 38092368 10.1093/jisesa/iead112 PMC10718815

[49] D. Task, C.-C. Lin, A. Vulpe, A. Afify, S. Ballou, M. Brbic, P. Schlegel, J. Raji, G. S. Jefferis, H. Li, K. Menuz, C. J. Potter, Chemoreceptor co-expression in *Drosophila melanogaster* olfactory neurons. eLife 11, e72599 (2022). 35442190 10.7554/eLife.72599 PMC9020824

[50] A. F. Silbering, R. Rytz, Y. Grosjean, L. Abuin, P. Ramdya, G. S. X. E. Jefferis, R. Benton, Complementary function and integrated wiring of the evolutionarily distinct *Drosophila* olfactory subsystems. J. Neurosci. 31, 13357–13375 (2011). 21940430 10.1523/JNEUROSCI.2360-11.2011 PMC6623294

[51] M. Ai, S. Blais, J.-Y. Park, S. Min, T. A. Neubert, G. S. B. Suh, Ionotropic glutamate receptors IR64a and IR8a form a functional odorant receptor complex in vivo in *Drosophila*. J. Neurosci. 33, 10741–10749 (2013). 23804096 10.1523/JNEUROSCI.5419-12.2013 PMC3693055

[52] J. Zhang, S. Bisch-Knaden, R. A. Fandino, S. Yan, G. F. Obiero, E. Grosse-Wilde, B. S. Hansson, M. Knaden, The olfactory coreceptor IR8a governs larval feces-mediated competition avoidance in a hawkmoth. Proc. Natl. Acad. Sci. U.S.A. 116, 21828–21833 (2019). 31591212 10.1073/pnas.1913485116 PMC6815144

[53] J. I. Raji, N. Melo, J. S. Castillo, S. Gonzalez, V. Saldana, M. C. Stensmyr, M. DeGennaro, *Aedes aegypti* mosquitoes detect acidic volatiles found in human odor using the IR8a pathway. Curr. Biol. 29, 1253–1262.e7 (2019). 30930038 10.1016/j.cub.2019.02.045 PMC6482070

[54] K. Vogt, D. M. Zimmerman, M. Schlichting, L. Hernandez-Nunez, S. Qin, K. Malacon, M. Rosbash, C. Pehlevan, A. Cardona, A. D. T. Samuel, Internal state configures olfactory behavior and early sensory processing in *Drosophila* larvae. Sci. Adv. 7, eabd6900 (2021). 33523854 10.1126/sciadv.abd6900 PMC7775770

[55] L. P. C. Lewis, K. P. Siju, Y. Aso, A. B. Friedrich, A. J. B. Bulteel, G. M. Rubin, I. C. Grunwald Kadow, A higher brain circuit for immediate integration of conflicting sensory information in *Drosophila*. Curr. Biol. 25, 2203–2214 (2015). 26299514 10.1016/j.cub.2015.07.015

[56] Y. Wang, Y. Pu, P. Shen, Neuropeptide-gated perception of appetitive olfactory inputs in *Drosophila* larvae. Cell Rep. 3, 820–830 (2013). 23453968 10.1016/j.celrep.2013.02.003

