# Supplementary material for: Multimodal social context modulates larval behavior in Drosophila
Source: Sci Adv. 2026 Jan 30;12(5):eady0750. doi: 10.1126/sciadv.ady0750 (PMC12857689; doi:10.1126/sciadv.ady0750)
Supplement: Supplementary file 1 — Figs. S1 to S8 Table S1 Legends for movies S1 to S5 Legends for data S1 to S3 [file sciadv.ady0750_sm.pdf]

Science Advances
AAAS

Supplementary Materials for

Multimodal social context modulates larval behavior in *Drosophila*

Akhila Mudunuri *et al.*

Corresponding author: Katrin Vogt, katrin.vogt@uni-konstanz.de

Sci. Adv. **12**, eady0750 (2026)
DOI: 10.1126/sciadv.ady0750

The PDF file includes:

Figs. S1 to S8
Table S1
Legends for movies S1 to S5
Legends for data S1 to S3

Other Supplementary Material for this manuscript includes the following:

Movies S1 to S5
Data S1 to S3

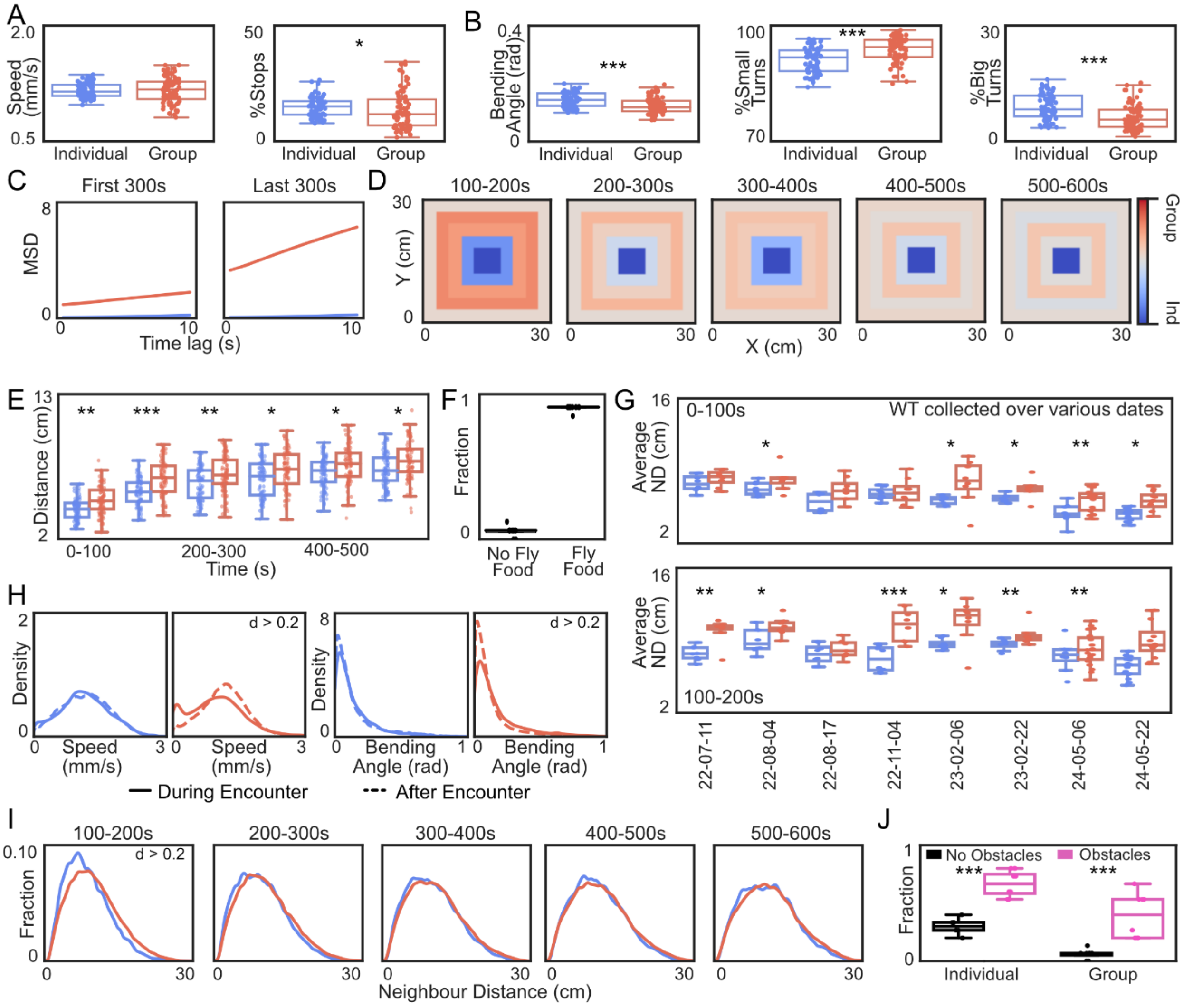

Fig. S1: Behavioral analysis for larvae as individuals and in a group: (**A**) Run speed and percentage of stops for the first 100s for superimposed groups (blue) and real groups (red). (**B**) Bending angle, percentage of small turns and big turns for the first 100s for superimposed groups (blue) and real groups (red). (**C**) Mean Squared Displacement (MSD) for real and superimposed groups for the first 300s and last 300s of the experiment. (**D**) Spatial distribution heatmaps for WT data from 100-600s of the experiment in 100s intervals. (**E**) Distance from the center for real or superimposed individual groups over time. (**F**) Fraction of larvae in the center with food or no food placed in the center of the arena (N = 8 trials). (**G**) The average neighbor distance of superimposed individual groups (blue) and real groups (red) for WT data collected over various time points. (**H**) Speed and bending angle for superimposed groups (blue) and real groups (red) during (line) and for 5s after encounter (dashed line). (effect size = Cohen's d ($d < 0.2$ – negligible, $0.2 < d < 0.5$ – small, $0.5 < d < 0.8$ – moderate, $d > 0.8$ – high)) (**I**) Neighbor distance for WT data from 100-600s of the experiment in 100s intervals. (**J**) Fraction of larvae in the center for superimposed individual larvae (bootstrapped to N=8 trials) and groups of 15 larvae (N = 8) in the absence (black) and presence of obstacles (pink). (Bootstrapped CI test for individuals and Mann-Whitney U test for groups = * $p < 0.05$; ** $p < 0.01$, *** $p < 0.001$).

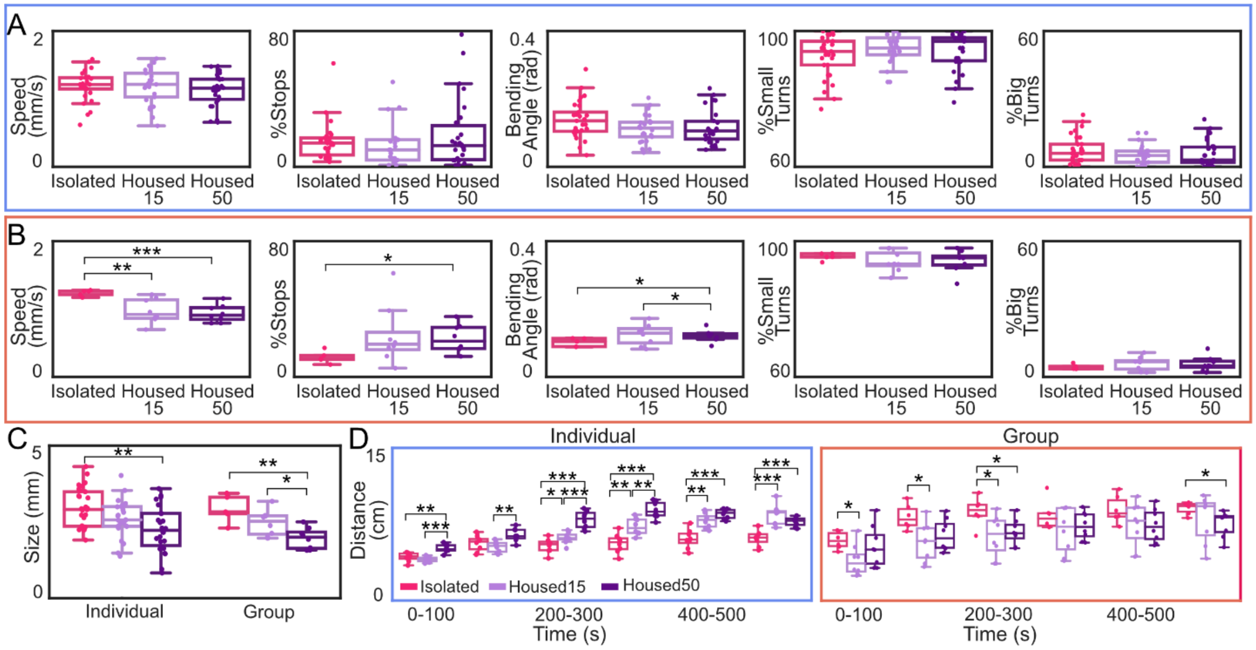

Fig. S2: Behavioral analysis for larvae with different developmental experiences as individuals and in groups: (**A**) Speed, percentage of stops, bending angle, percentage of small turns and big turns for the first 100s for individuals of isolated (pink), housed15 (light purple), and housed50 (dark purple) conditions. (**B**) Speed, percentage of stops, bending angle, percentage of small turns, and big turns for the first 100s for groups of isolated (pink), housed15 (light purple), and housed50 (dark purple) conditions. (**C**) Size of individual larvae and larvae in groups. (**D**) Average distance from the center over time for superimposed groups and real groups. (Mann-Whitney U test = * $p < 0.05$; ** $p < 0.01$, *** $p < 0.001$)

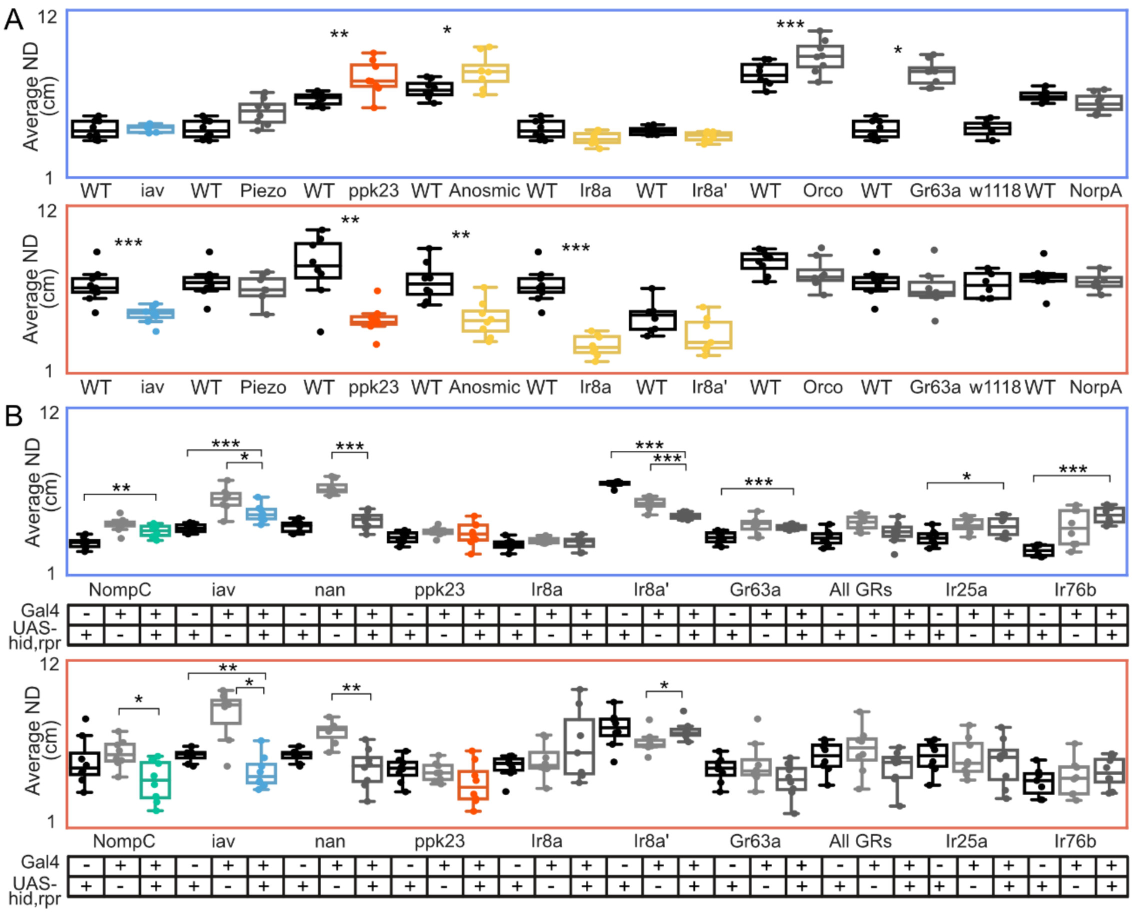

Fig. S3: Summary of all genetic screenings for the superimposed individual groups with social phenotype: (**A**) Mutant experiments: Superimposed individual groups (blue box) and real groups (red box) for the first 100s. WT control (black, N = 8) and mutant lines with no phenotype (grey, N=8) and with phenotype (different colors, N = 8). (**B**) Genetic silencing: Superimposed individual groups (blue box) and real groups (red box) for the first 100s. *WT x hid,reaper* (black, N = 8), experimental controls/GAL4 controls with no phenotype (grey, N = 8), and cross hits (different colors, N = 8). (Bootstrapped CI test for individuals and Mann-Whitney U test for groups = * $p < 0.05$; ** $p < 0.01$, *** $p < 0.001$)

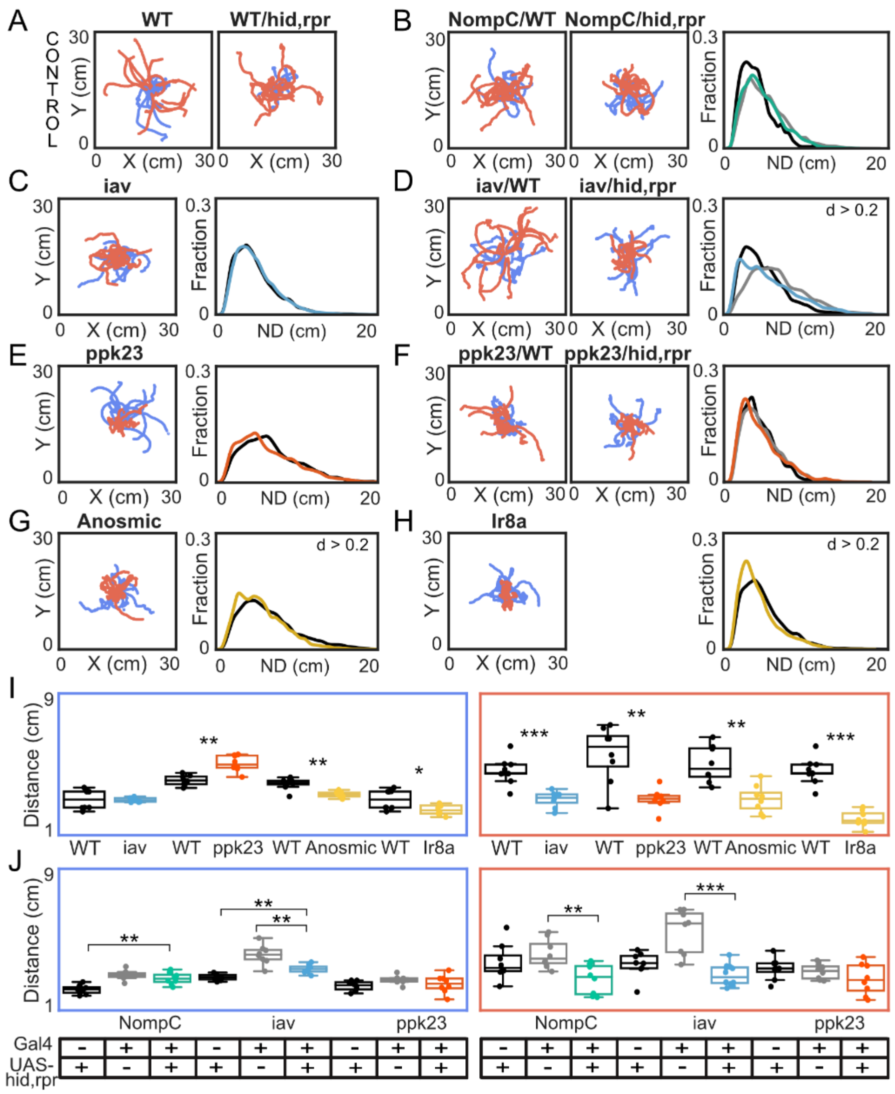

Fig. S4: Behavioral analysis for the superimposed individual groups with social phenotype: (**A**) Sample trajectories of WT and *WT x hid,reaper* controls for a superimposed individual group (blue) and real group (red). (**B-H**) Sample trajectories. Neighbor distance for the first 100s in all superimposed individual groups. Effect size = Cohen's d ($0.2 < d < 0.5$ – small). (**I**) Distance from the center for mutant experiments: Superimposed individual groups (blue box) and real groups (red box) for the first 100s. WT control (black, N = 8) and with phenotype (different colors, N = 8). (**J**) Distance from the center for genetic silencing: Superimposed individual groups (blue box) and real groups (red box) for the first 100s. *WT x hid,reaper* (black, N = 8), GAL4 controls with no phenotype (grey, N = 8), and cross hits (different colors, N = 8). (Bootstrapped CI test for individuals and Mann-Whitney U test for groups = * $p < 0.05$; ** $p < 0.01$, *** $p < 0.001$)

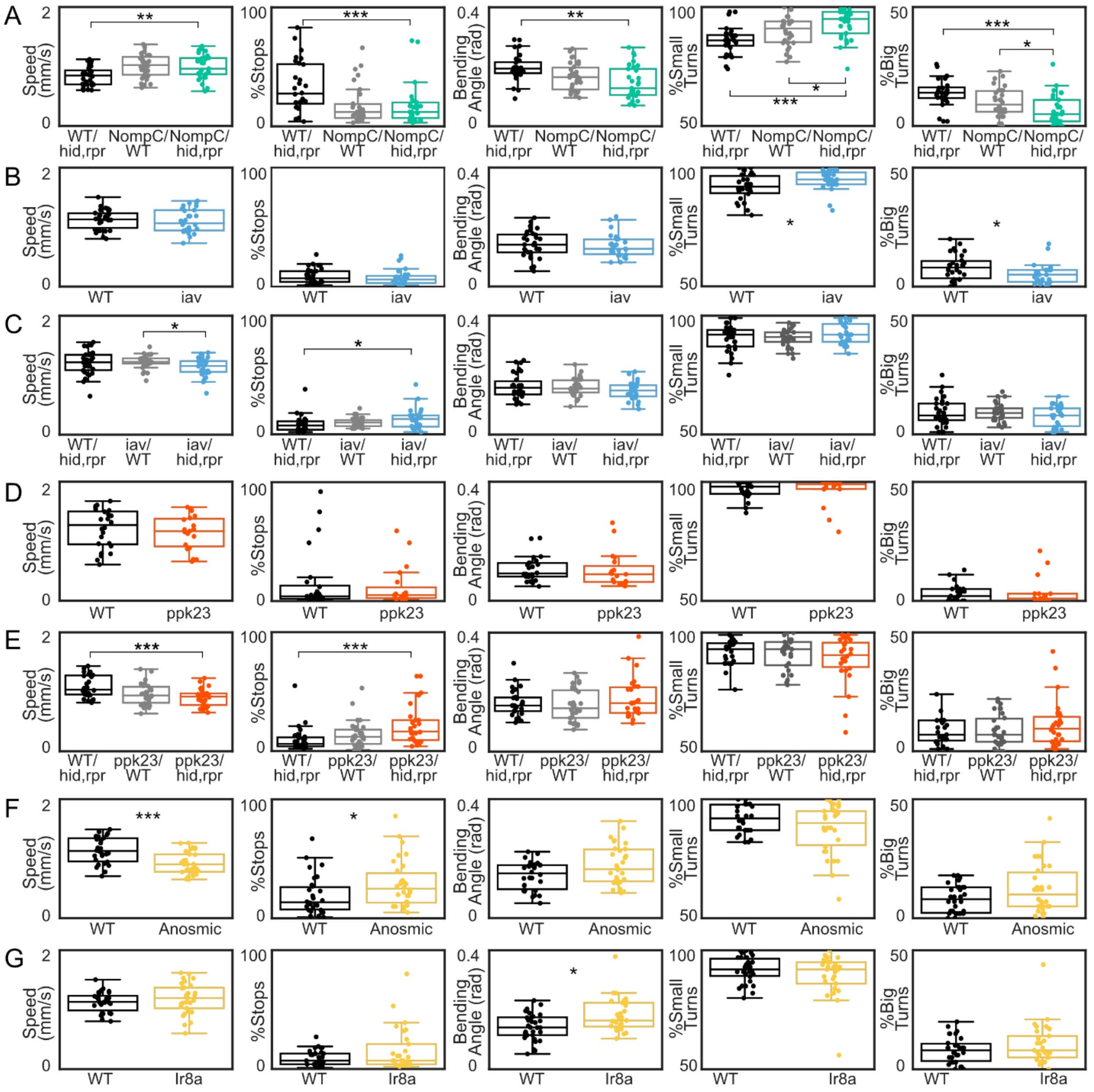

Fig. S5: Behavioral analysis for individuals with social phenotype: (**A-G**) Speed, percentage of stops, bending angle, percentage of small and big turns of individuals for mutant experiments (WT control (black, N = 30) and mutant lines with phenotype (different colors, N = 30)) and for genetic silencing experiments (*WT x hid,reaper* (black, N = 30), GAL4 controls with no phenotype (grey, N = 30), and cross hits (different colors, N = 30)). (Mann-Whitney U test = * $p < 0.05$; ** $p < 0.01$, *** $p < 0.001$)

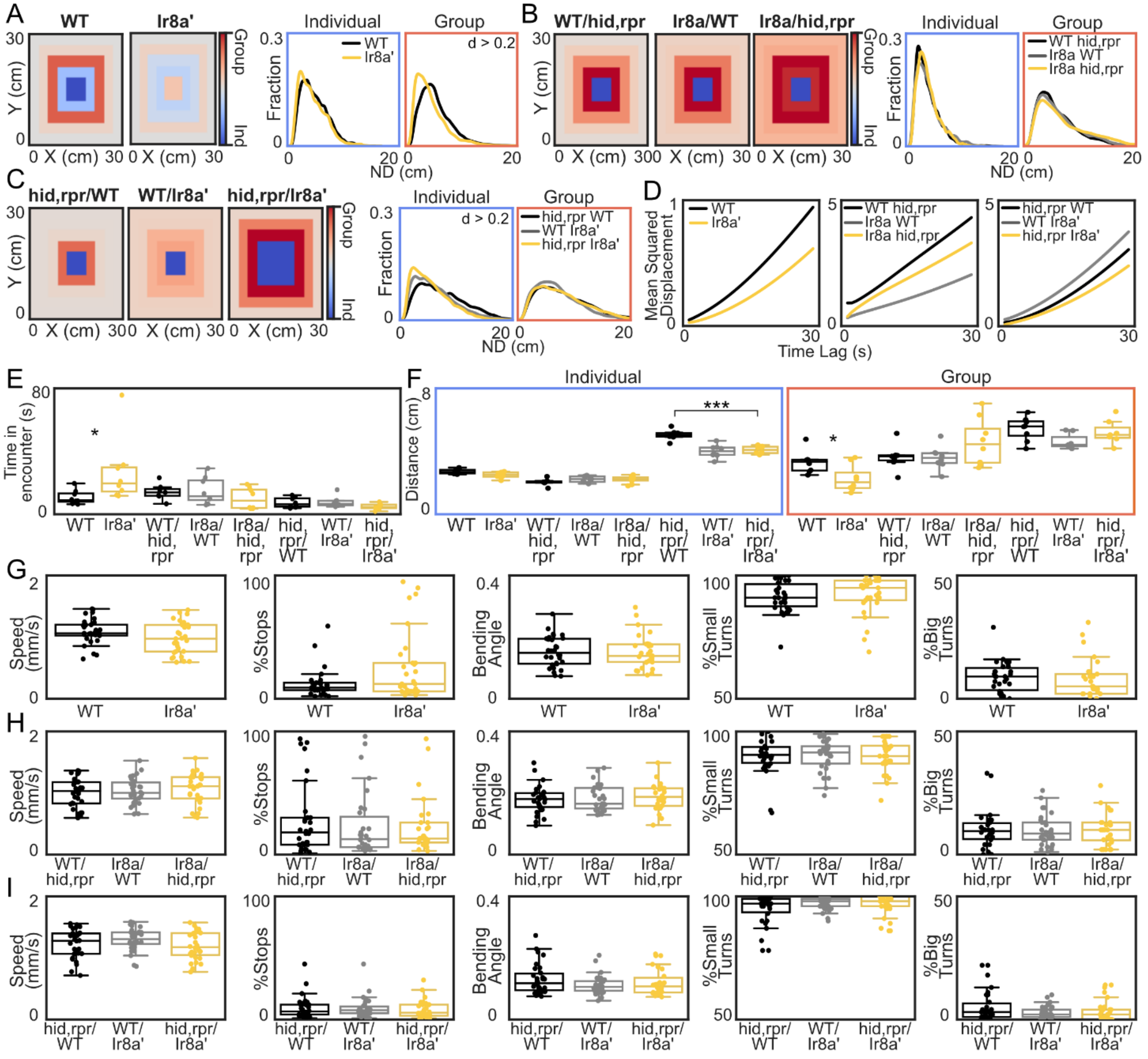

Fig. S6: Behavioral analysis for Ir8a: (**A**) Distribution heatmaps for WT (N = 8) and Ir8a'mutants (N=8) for the first 100s. Neighbor distance for the first 100s in all superimposed individual groups and real groups. WT = black, Ir8a' = yellow (**B**) Distribution heatmaps for WT/hid,rpr (N = 8), Ir8a/WT (N=8), and Ir8a/hid,rpr (N=8) for the first 100s. Neighbor distance for the first 100s in all superimposed individual groups and real groups. Controls = black/gray, Ir8a/hid,rpr = yellow. (**C**) Distribution heatmaps for hid,rpr/WT (N = 8), WT/Ir8a' (N=7), and hid,rpr/Ir8a' (N=7) for the first 100s. Neighbor distance for the first 100s in all superimposed individual groups and real groups. Controls = black/gray, hid,rpr/Ir8a' = yellow. (**D**) Mean squared displacement for the first 100s for real groups. (**E**) Time in encounters for experimental controls and treatments. (**F**) Distance from center for the first 100s for controls and treatments in superimposed individual groups and real groups. (Bootstrapped CI test for individuals and Mann-Whitney U test for groups = * $p < 0.05$; ** $p < 0.01$, *** $p < 0.001$) (**G-I**) Speed, percentage of stops, bending angle, percentage of small and big turns for individuals. (Mann-Whitney U test = * $p < 0.05$; ** $p < 0.01$, *** $p < 0.001$)

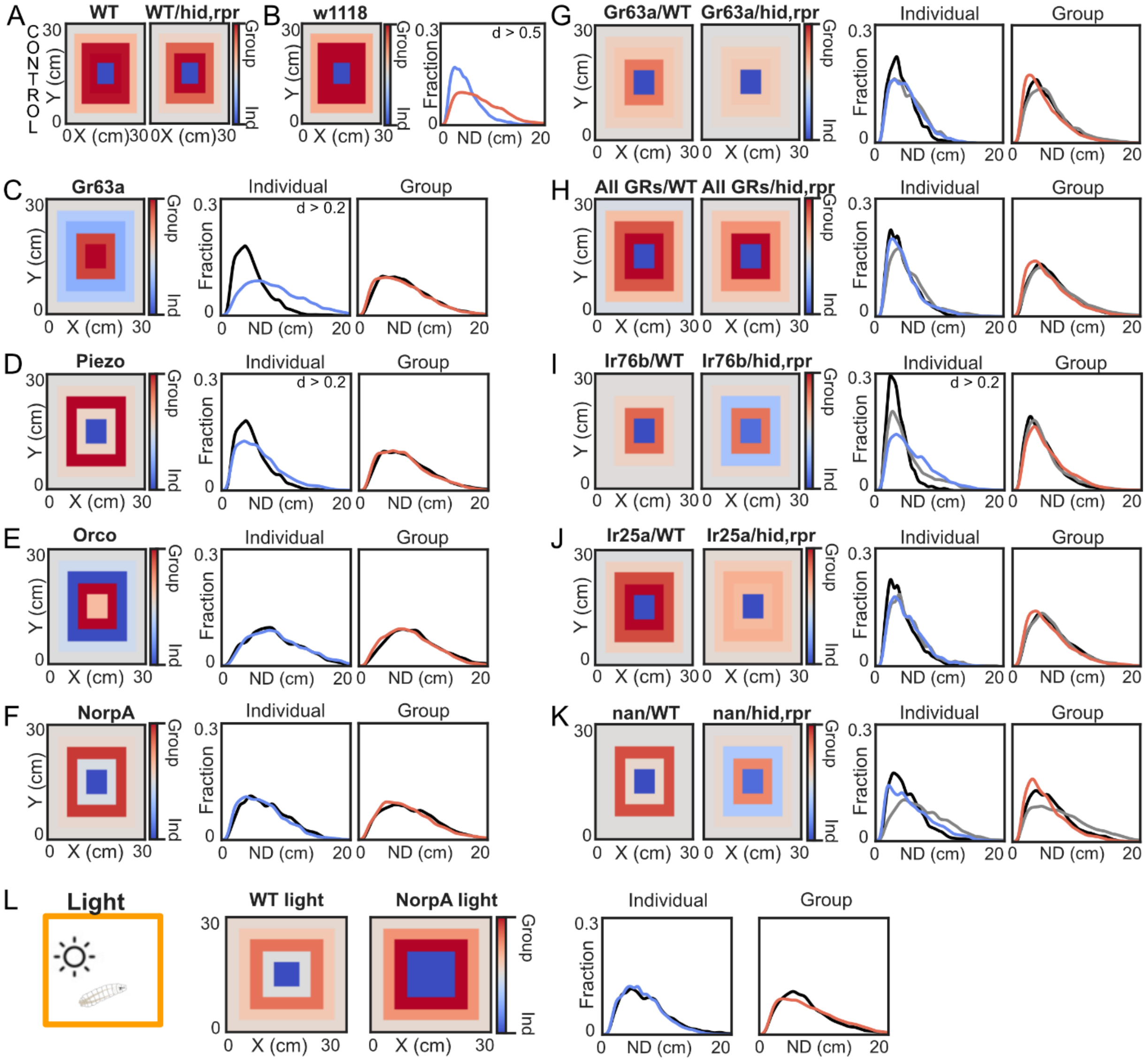

Fig. S7: Behavioral data for the genetic experiments with no phenotype: (**A**) Distribution heatmaps for the pooled controls: WT (N = 32) and *WT x hid,reaper* (N = 32). (**B**) Distribution heatmap and neighbor distance for the first 100s of superimposed individual groups and real groups for w1118 larvae (N = 8). Effect size = Cohen's d (0.2 < d < 0.5 – small, 0.5 < d < 0.8 – moderate). Distribution heatmap for w1118 larvae. (**C-K**) Distribution heatmaps for all controls and treatment groups. Neighbor distance for the first 100s in all superimposed individual groups and real groups. Controls = black/gray, Experimental groups = blue (superimposed individual group) / red (real group). (**L**) Distribution heatmaps for WT and NorpA tested in the presence of light (N = 8). Neighbour distance for the first 100s for superimposed individual groups and real groups. WT = black, NorpA = blue (superimposed individual group) / red(real group).

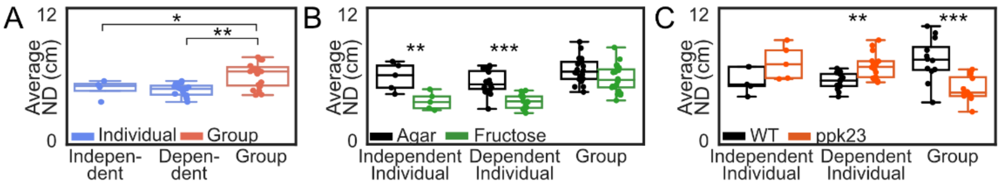

Fig. S8: Bootstrapped data for a subset of the experiments for an independent and dependent individual dataset: (**A**) Average neighbor distance of superimposed individual groups from independent (blue) and dependent (blue) datasets compared to real groups (red) for WT data collected over various time points. (Bootstapped CI test = * $p < 0.05$; ** $p < 0.01$, *** $p < 0.001$). (Bootstrapped CI test = * $p < 0.05$; ** $p < 0.01$, *** $p < 0.001$) (**B**) Average neighbor distance of superimposed individual groups from independent and dependent datasets compared to real groups for agar (black) and fructose (green) from Fig 2. (**C**) Average neighbor distance of superimposed individual groups from independent and dependent datasets compared to real groups for WT (black) and ppk23 mutant (orange) from Fig 4. (Bootstrapped CI test for individuals and Mann-Whitney U test for groups = * $p < 0.05$; ** $p < 0.01$, *** $p < 0.001$)

Genotype	Source
UAS-hid,reaper	Kindly provided by the Thum lab (32)
$iav^{-/-}$	BDSC #24768
Piezo [KO]	BDSC #58770
Δppk23	BDSC #33300
Anosmic	Kindly provided by the Galizia lab (23)
Orco [1]	BDSC #23129
Gr63a [1]	BDSC #9941
Ir8a [1]	BDSC #41731
Ir8a’	BDSC #93790
NorpA	BDSC #9047
w1118	BDSC #3605
NompC-Gal4	BDSC #36361
iav-Gal4	BDSC #52273
nan-Gal4	BDSC #24903
ppk23-Gal4	BDSC #93026
Ir8a-Gal4	BDSC #41731
Gr63a-Gal4	BDSC #9942
All GRs-Gal4	Kindly provided by the Sprecher lab (GO-Gal4) (37)
Ir25a-Gal4	BDSC #41728
Ir76b-Gal4	BDSC #41730

Table S1. Fly lines used in this study.

Supplementary Videos

Movie S1.
Dispersal behavior of larvae in a group of 15 on agar for the first 100s.
Movie S2.
Movie of a group of 15 larvae on agar for 600s (10x speed) with TRex tracking software in the 25x25 cm assay.
Movie S3.
Dispersal behavior of an individual larva on agar for the first 100s.
Movie S4.
Dispersal behavior of larvae in a group of 15 exposed to standard cornmeal food for the first 100s.
Movie S5.
Dispersal behavior of individual larvae exposed to agar obstacles for the first 100s.

Supplementary Excel Files

Data S1.
Statistical results for all data in the main and supplement figures.
Data S2.
Locomotion parameter values for all experiments (Average +/- STD).
Data S3.
Statistics for MSD analysis (D-values).
